# Schottky Junctions with Bi@Bi_2_MoO_6_ Core-Shell Photocatalysts toward High-Efficiency Solar N_2_-to-Ammonnia Conversion in Aqueous Phase

**DOI:** 10.3390/nano14090780

**Published:** 2024-04-30

**Authors:** Meijiao Wang, Guosong Wei, Renjie Li, Meng Yu, Guangbo Liu, Yanhua Peng

**Affiliations:** 1 College of Chemistry and Chemical Engineering, Qingdao University, Qingdao 266071, China; 15753740944@163.com (M.W.); 18563620407@163.com (G.W.); lrj222629@163.com (R.L.); yumeng1@qdu.edu.cn (M.Y.); 2 Key Laboratory of Biofuels, Qingdao Institute of Bioenergy and Bioprocess Technology, Chinese Academy of Sciences, Qingdao 266101, China

**Keywords:** nitrogen reduction reaction, Schottky junction, core-shell structure, localized surface plasmon resonance, Bi@Bi_2_MoO_6_

## Abstract

The photocatalytic nitrogen reduction reaction (NRR) in aqueous solution is a green and sustainable strategy for ammonia production. Nonetheless, the efficiency of the process still has a wide gap compared to that of the Haber–Bosch one due to the difficulty of N_2_ activation and the quick recombination of photo-generated carriers. Herein, a core-shell Bi@Bi_2_MoO_6_ microsphere through constructing Schottky junctions has been explored as a robust photocatalyst toward N_2_ reduction to NH_3_. Metal Bi self-reduced onto Bi_2_MoO_6_ not only spurs the photo-generated electron and hole separation owing to the Schottky junction at the interface of Bi and Bi_2_MoO_6_ but also promotes N_2_ adsorption and activation at Bi active sites synchronously. As a result, the yield of the photocatalytic N_2_-to-ammonia conversion reaches up to 173.40 μmol g^−1^ on core-shell Bi@Bi_2_MoO_6_ photocatalysts, as much as two times of that of bare Bi_2_MoO_6_. This work provides a new design for the decarbonization of the nitrogen reduction reaction by the utilization of renewable energy sources.

## 1. Introduction

Ammonia (NH_3_) is one of the most essential fundamental industrial chemicals owing to its roles in carbon-free energy storage and the production of fertilizer [1,2,3]. The Haber–Bosch process for industrial synthetic ammonia requires greatly harsh conditions operating at a very high temperature (≈700 K) and very high pressures (≈100 atm), which result in great energy consumption and environment-harmful gas emissions [4,5,6]. Compared to the conventional process, the photocatalytic nitrogen reduction reaction (NRR) in aqueous solution offers a green and sustainable technology for ammonia production, which promotes widespread investigations [7,8,9,10]. However, the efficiency of this process still has a wide gap compared to that of the Haber–Bosch one due to the difficulty of N_2_ activation and the quick recombination of photo-generated carriers [11,12,13]. The strategies of innovative fabrication for functional material design, including defect and dopant engineering, heterojunction construction by integrating a semiconductor and metal or another semiconductor, reactive crystal facet exposure and so on, have been widely explored [14,15,16,17]. The purpose mainly focuses on the functionality that not only facilitates the separation of photo-excited electrons and holes but also provides more N_2_ activation sites for solar-to-ammonia conversion.

Among numerous photocatalysts, bismuth molybdate (Bi_2_MoO_6_) has been widely employed for photocatalytic nitrogen fixation due to the low cost, environmentally friendly characteristics, excellent thermal and chemical stability and tunable physical and electronic properties [18,19]. As the simplest member of the Aurivillius oxide family, Bi_2_MoO_6_ possesses a fascinating structure that is composed of [Bi_2_O_2_]^2+^ slices linking with a corner-sharing structure of MoO_6_ octahedra [20]. The layered configuration decides the visible light response and facilitates good electron conductivity. However, Bi_2_MoO_6_ also faces the same problems as all photocatalysts that are the sluggish transportation and the rapid recombination of photo-generated carriers, which leads to the low photocatalytic efficiency for the NRR. The formation of a Schottky junction by rectifying contact between semiconductors and metals can facilitate the separation of photo-generated carriers and trap the electrons on metals due to the low-lying Fermi level (*E*_F_) [21,22]. Thus, tremendous efforts have been devoted to suppressing external electron–hole recombination and maximizing the utilization of incident photons. Some noble metals (Au, Ag, etc.) loading on the Bi_2_MoO_6_ surface to form Schottky barriers and excite the localized surface plasmon resonance (LSPR) synchronously has been proven to be an efficient strategy [23,24,25,26,27]. However, the Schottky junctions’ directed charge transfer results in the depletion of electrons at Bi_2_MoO_6_, which is disadvantageous for N_2_ adsorption and activation dominated by electron transfer. In addition, loaded noble metals always become the active centers for concomitant H_2_ production due to the weak metal hydrogen strength, thereby suppressing the NRR.

Metal bismuth (Bi) is an ideal choice to take the place of noble metals due to its lower cost and strong plasmonic effect [28,29,30]. Most importantly, Bi can be easily self-reduced onto Bi_2_MoO_6_ by an in situ solvothermal process with its unique advantages of the intimate interfacial contact between Bi and Bi_2_MoO_6_ due to the same containing elements [31,32,33]. Theoretical studies by Norskov and coworkers reported the low HER activity of Bi due to the highest hydrogen binding energy (~0.75 eV) [34]. Moreover, it is found that N_2_ preferably adsorbs at Bi sites with an end-on bound structure, which suggests favorable N_2_ adsorption and activation at Bi sites [35,36]. Recently, many investigations have proved that Bi/semiconductors possess obviously enhanced photocatalytic activity in the NRR [37,38]. Huang et al. deposited Bi nanoparticles on BiOBr to construct a Schottky junction and found greatly improved photocatalytic NRR performance in an aqueous reaction [22]. However, the Schottky junction has a limited interface contact as compared with the core-shell structure. In our previous studies, it was found that the formation of core-shell heterojunctions makes obviously enhanced photocatalytic activity [39,40,41]. Therefore, the core-shell Bi@Bi_2_MoO_6_ Schottky junction on a highly efficient photocatalytic NRR can be expected.

Herein, a core-shell Bi@Bi_2_MoO_6_ microsphere through constructing Schottky junctions is explored as a robust photocatalyst toward N_2_ reduction to ammonia. Metal Bi self-reduced onto Bi_2_MoO_6_ not only spurs the photo-generated electron and hole separation owing to the Schottky junction at the interface of Bi and Bi_2_MoO_6_ but also promotes N_2_ adsorption and activation at Bi active sites synchronously. As a result, the yield of the photocatalytic N_2_-to-ammonia conversion reaches up to 173.40 μmol g^−1^ on core-shell Bi@Bi_2_MoO_6_ photocatalysts, as much as two times of that of bare Bi_2_MoO_6_.

## 2. Experimental Section

### 2.1. Materials

All of the chemical reagents involving bismuth nitrate pentahydrate (Bi(NO_3_)_3_·5H_2_O), sodium molybdate dihydrate (Na_2_MoO_4_·2H_2_O), ethylene glycol (EG), ethanol absolute (C_2_H_5_OH), sodium hydroxide(NaOH) and ammonium chloride (NH_4_Cl) are of analytical grade without any additional purification.

### 2.2. Preparation of Photocatalysts

Synthesis of Bi_2_MoO_6_: Bi_2_MoO_6_ microspheres were prepared by a simple solvothermal method [24]. Typically, 2.0 mmol of Bi(NO_3_)_3_·5H_2_O and 1.0 mmol of Na_2_MoO_4_·2H_2_O were dissolved in 30 mL of ethylene glycol (EG), respectively. Then, the mixture was stirred vigorously until a clear solution was formed. Subsequently, the sodium molybdate solution was slowly dripped into the bismuth nitrate solution and stirred for 30 min at room temperature. The obtained solution was thermally treated at 160 °C for 8 h in a 100 mL Teflon-lined stainless-steel autoclave. After filtering and thoroughly washing with deionized water and absolute ethanol, the Bi_2_MoO_6_ products were obtained by drying the material at 60 °C for 12 h. The product was labeled as BMO.

Synthesis of Bi@Bi_2_MoO_6_: Core-shell Bi@Bi_2_MoO_6_ materials were prepared using the same process as Bi_2_MoO_6_ photocatalysts, apart from the solvothermal reaction time. The reaction times were prolonged to 12 h, 16 h, 20 h, 24 h, 28 h and 32 h to obtain Bi@Bi_2_MoO_6_ photocatalysts, labeled as B@BMO-1, B@BMO-2, B@BMO-3, B@BMO-4, B@BMO-5 and B@BMO-6, respectively.

### 2.3. Characterization of Photocatalysts

The phase compositions of the samples were examined by powder XRD (Smart Lab 3 KW). A scanning electron microscope (SEM) (Sigma500, Carl Zeiss AG, Oberkochen, Germany) and transmission electron microscope (TEM) with an electron acceleration energy of 200 kV (JEM 2100F, JEOL, Tokyo, Japan) were employed to assess the morphology and structure. The chemical states and surface compositions of the samples were determined using an X-ray photoelectron spectrometer (ESCALAB Xi+, Thermo Fisher Scientific, Waltham, MA, USA), and the C 1s peak at 284.8 eV was used as a reference to calibrate the peak positions, and the peaks were fitted using Avantage. The UV-vis absorption spectra were acquired for the dry-pressed disk samples using a Scan UV-vis spectrophotometer, applying pure BaSO_4_ as the reflectance sample. Time-resolved PL decay curves were obtained using a FLS980 fluorescence lifetime spectrophotometer (Edinburgh Instruments, Edinburgh, UK), and the decay curves were fitted to a triple-exponential model. Inductively coupled plasma mass spectrometry (ICP-MS) was used to determine the metal content.

### 2.4. Photoelectrochemical Measurements

Photoelectrochemical tests were performed on an electrochemical workstation (Model CHI 760D, CH instruments, Inc., Austin, TX, USA). The workstation was connected to a three-electrode system consisting of a working electrode, a counter electrode (Pt sheet) and a reference electrode (Ag/AgCl). To prepare the working electrode, 10 mg of photocatalysts, 10 μL of Trillatone X-100, 20 μL of acetylacetone and 80 μL of deionized water were ground and mixed well to coat the marked area (1 cm × 1 cm) on a piece of 2 cm × 1 cm FTO glass. The electrode was calcined at 200 °C for 2 h. The transient photocurrent responses were carried out in Na_2_SO_4_ aqueous solution under 300 W Xe lamp irradiation without any filters. Electrochemical impedance spectroscopy (EIS) was carried out in the frequency range of 0.005~10000 Hz. The potential (vs. Ag/AgCl) was adjusted to (vs. NHE) by the equation as follows [37]:

E (vs. NHE) = E (vs. Ag/AgCl) + 0.197 V + 0.0591 × pH(1)

### 2.5. Photocatalytic N_2_ Fixation Reaction

The photocatalytic N_2_ reduction reaction was carried out under mild conditions. A total of 10 mg of photocatalyst was dispersed in 25 mL of DI water ultrasonically for 10 min, and then the suspension liquid was transferred to a photocatalytic quartz reactor to catalyze the N_2_ reduction reaction. The suspension was vigorously stirred in the dark for 30 min, and high-purity N_2_ (100 mL min^−1^) was bubbled to eliminate dissolved oxygen and saturate dissolved N_2_. The 300 W Xe lamp was used as a simulated light source. During the irradiation process, 3 mL of the solution was taken out every 30 min and filtered through a 0.22 µm MCE membrane to remove the photocatalyst. The concentration of ammonia (NH_4_^+^) was detected using the indophenol blue method at 655 nm on a UV-Vis spectrophotometer.

### 2.6. Determination of Ammonia

The amount of NH_3_ in the reaction solution was determined using the indophenol blue method. Specifically, 2 mL of 1 M NaOH solution was added to the solution containing salicylic acid and sodium citrate. After that, 1 mL of 0.05 M NaClO and 0.2 mL of the C_3_FeN_6_Na_2_O solution (1%wt) were sequentially added to 2 mL reaction solution. Placing at room temperature for 30 min, the NH_3_ concentration was determined through the standard curve of NH_3_ using a UV-Vis absorption spectrometer, measuring the absorbance at 655 nm.

## 3. Results and Discussion

### 3.1. The Structure of Samples

**Figure 1:**
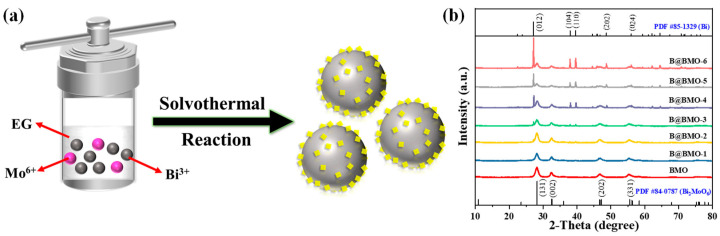
(**a**) Schematics of preparation of core-shell Bi@Bi_2_MoO_6_ composites; (**b**) XRD patterns of Bi_2_MoO_6_ materials and Bi@Bi_2_MoO_6_ composites.

As a photoactive semiconductor, Bi_2_MoO_6_ nanosheets have been prepared for the construction of Schottky junction photocatalysts. In such a reaction system, ethylene glycol is a reducing agent. Through controlling the solvothermal reaction time, Bi^3+^ ions are in situ reduced to the metal Bi microsphere as a core, while Bi_2_MoO_6_ nanosheets are loaded onto the surface of the Bi microsphere as the shell (Figure 1a). The core-shell Bi@Bi_2_MoO_6_ composite in situ growth not only solves the problems that resulted from foreign elements but also benefits the formation of an intimate interface between Bi and Bi_2_MoO_6_, which would accelerate the separation and transfer of photo-generated electrons and holes, thereby promoting photocatalytic activity. XRD patterns are used to examine the crystalline phase and composition of composites (Figure 1b). The diffraction peaks of pure Bi_2_MoO_6_ are perfectly indexed as orthorhombic Bi_2_MoO_6_ (PDF 84-0787). For the Bi@Bi_2_MoO_6_ composites, additional diffraction peaks which are derived from metal Bi (PDF 85-1329) are obviously discernable, suggesting the successful preparation of Bi@Bi_2_MoO_6_ composites. A possible growth mechanism has been proposed as similar as the previous report [42]. Firstly, the hydrolysis reaction of [MoO_4_]^2−^ into H_2_MoO_4_ occurs (Equation (2)). Then, it would react with Bi^3+^ in the solution to form Bi_2_MoO_6_ through an ion-exchange process (Equation (3)), which is the reason why only Bi_2_MoO_6_ is observed in the reaction time less than 12 h. By increasing the reaction time, the diffraction characteristic peaks associated with metal Bi (012), (104) and (110) can be clearly observed, and the diffraction peak intensity of metal Bi gradually increases, which indicates that Bi_2_MoO_6_ is in situ reduced to metal Bi by ethylene glycol (Equation (4)). The results of XRD confirm our predictions as well as the reaction process.

[MoO_4_]^2−^ + 2H_2_O → H_2_MoO_4_ + 2OH^−^ (2)

H_2_MoO_4_ + 2Bi^3+^ + 2H_2_O → Bi_2_MoO_6_ + 6H^+^ (3)

Bi_2_MoO_6_ + C_2_H_6_O_2_ → 2Bi + C_2_H_2_O_4_ + H_2_ + H_2_MoO_4_ (4)

**Figure 2:**
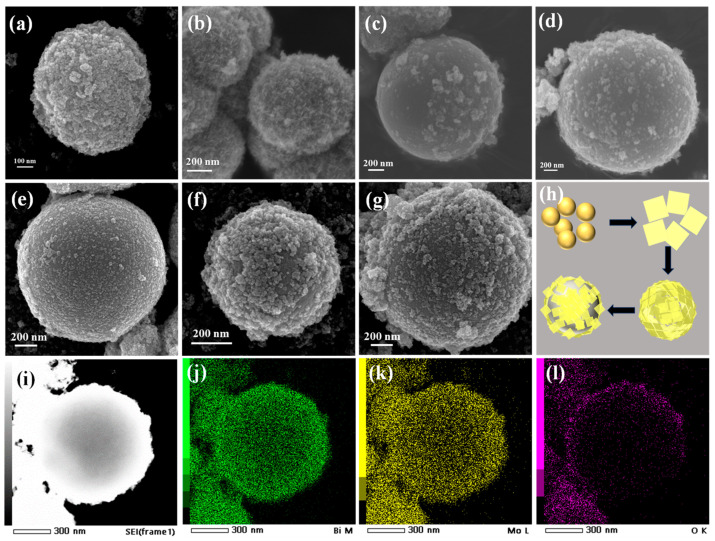
SEM images of BMO (**a**), B@BMO-1 (**b**), B@BMO-2 (**c**), B@BMO-3 (**d**), B@BMO-4 (**e**), B@BMO-5 (**f**) and B@BMO-6 (**g**), and the schematic diagram of the growth mechanism (**h**), and the elemental mapping images of Bi, Mo and O for the Bi@Bi_2_MoO_6_ composite (**i**–**l**).

The SEM is used to check the morphology of Bi_2_MoO_6_ materials and Bi@Bi_2_MoO_6_ composites. As shown in Figure 2a, Bi_2_MoO_6_ presents a typical microsphere-like structure with a diameter of 700 nm, which is formed by the accumulation of nanoparticles. For B@BMO-1, nanoparticles become nanosheets due to the Ostwald ripening process, while the morphology still keeps the microsphere structure (Figure 2b). At this stage, the crystal is mainly composed of Bi_2_MoO_6_, which agrees well with the results of XRD. Differently, B@BMO-2 exhibits a typical core-shell structure with a smooth sphere as the core and nanosheets as the shell (Figure 2c). According to the results, it can be concluded that the smooth sphere may be the metal Bi and the nanosheets may be the Bi_2_MoO_6_ due to the different morphology. Additionally, the longer the reaction time, the bigger the Bi spheres (Figure 2d–g). As shown in Figure 2h, a possible growth process of the Bi@Bi_2_MoO_6_ composite is considered, which is in agreement with the results of XRD and reaction equations. The related energy-dispersive X-ray (EDX) elemental mapping measurements are also investigated to determine the element distribution. The results are shown in Figure 2i–l, which indicate that Bi_2_MoO_6_ nanosheets are uniformly distributed on the surface of the Bi sphere, further demonstrating the formation of core-shell Bi@Bi_2_MoO_6_ composites.

**Figure 3:**
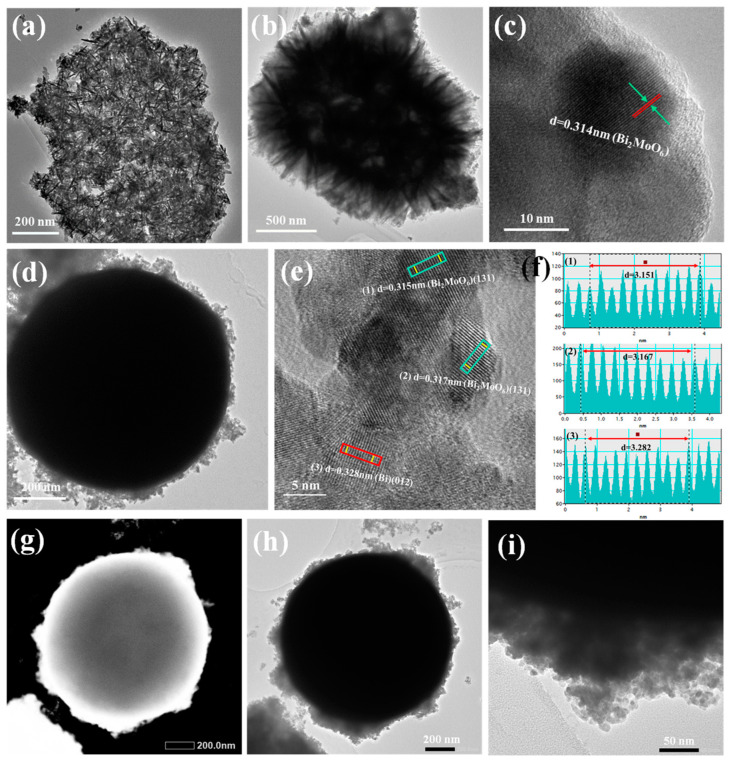
TEM and HRTEM images of BMO (**a**–**c**), B@BMO-4 (**d**–**f**) and B@BMO-6 (**g**–**i**).

To further determine the core-shell structure of Bi@Bi_2_MoO_6_ photocatalysts, the TEM and HRTEM images of Bi_2_MoO_6_ materials and Bi@Bi_2_MoO_6_ composites are shown in Figure 3. It can be found that pure Bi_2_MoO_6_ hierarchical structures are composed of ultrathin nanosheets with a thickness of ~20 nm and diameters in the range of 1~2 μm, which are shown in Figure 3a,b. From the high-magnification TEM images of Bi_2_MoO_6_ (Figure 3c), the clear lattice fringes with a spacing of 0.314 nm can be seen, which corresponds to the (131) crystal faces of Bi_2_MoO_6_ in the orthogonal phase. Figure 3d shows the TEM images of the B@BMO-4 sample. It can be observed that the very black smooth sphere with a diameter of about 1 μm is the core, and numerous ultrathin nanosheets with a transparent structure are stacked on the surface of the black sphere as the shell, which agrees with the results of the SEM. The results further confirm the successful formation of core-shell Bi@Bi_2_MoO_6_ composites. Moreover, the HRTEM images of the region with a green rectangle for the B@BMO-4 composite indicated the well-matched (131) plane of Bi_2_MoO_6_ with a lattice spacing of 0.315 and 0.314 nm (Figure 3e,f). The red rectangle for the B@BMO-4 composite corresponds to the (012) lattice plane of metal Bi particles with a lattice spacing of 0.328 nm, which is consistent with the XRD results. The TEM images of the B@BMO-6 sample are shown in Figure 3g,i; it can be found that the morphology of the B@BMO-6 sample is similar to that of B@BMO-4 with the core-shell structure which would have the benefit of photo-generated carrier separation and transfer.

**Figure 4:**
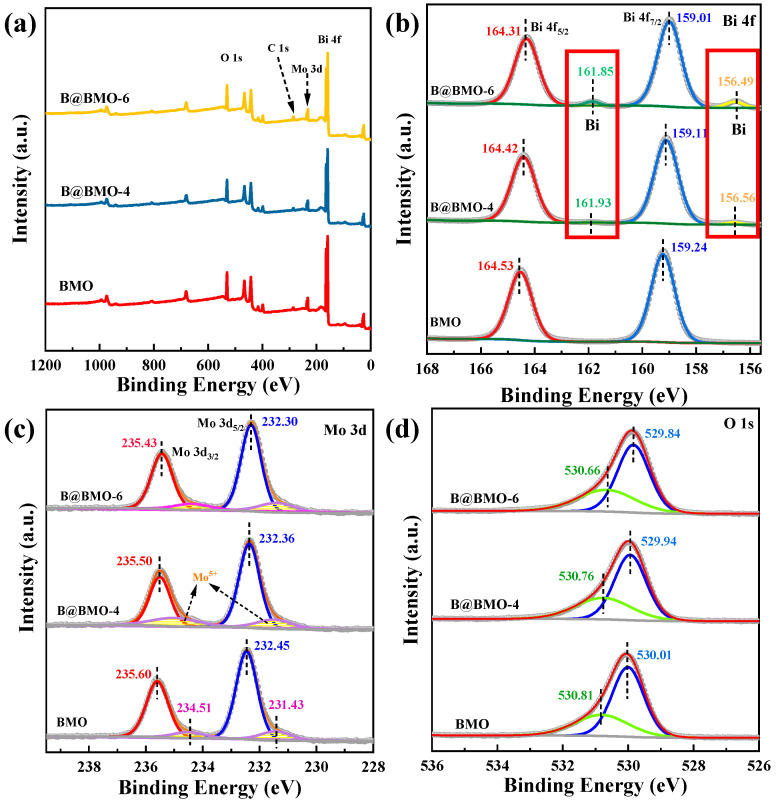
XPS spectra of BMO, B@BMO-4 and B@BMO-6 of survey (**a**), Bi 4f (**b**), Mo 3d (**c**) and O 1s (**d**).

To further understand the chemical compositions of BMO, B@BMO-4 and B@BMO-6, an X-ray photoelectron spectroscopy (XPS) measurement is carried out. As shown in Figure 4a, the surveys of three samples indicate that all the samples consist of Bi, Mo and O elements. All binding energy is calibrated with the C 1s band at 284.8 eV. The high-resolution XPS spectra of Bi 4f are displayed in Figure 4b. The binding energies located at 159.24 and 164.53 eV are attributed to Bi 4f_7/2_ and Bi 4f_5/2_, respectively, and no other bands can be observed, which demonstrated the existence of only Bi^3+^ for BMO samples [43]. Apart from the binding energies located at 159.11 and 164.42 eV that correspond to Bi 4f_7/2_ and Bi 4f_5/2_ of Bi^3+^, there are new two bands at 156.56 and 161.93 eV which are attributed to the metal Bi for B@BMO-4, suggesting the in situ formation of surface-detectable Bi due to the reduction reaction with the increase in reaction time. It can be seen that the intensity of bands at 156.49 and 161.85 eV increases for B@BMO-6, indicating more metal Bi is reduced. The Mo bands are divided into two bands at 232.45 and 235.60 eV assigned to Mo 3d_5/2_ and Mo 3d_3/2_, which are assigned to the bands of Mo^6+^ in Bi_2_MoO_6_ [44]. For B@BMO samples, there is a negative shift of about 0.1 eV in Mo 3d spectra as compared to that of BMO. In addition, the two bands of BMO at 231.43 and 234.51 eV are typical of Mo^5+^ cations, which indicates the introduction of some oxygen vacancies during metal Bi reduction [33]. Moreover, an obviously negative shift in Bi XPS spectra is also observed which suggests a strong interaction between metal Bi and Bi_2_MoO_6_ [45]. For the O 1s spectra (Figure 4d), two bands located at 530.01 and 530.81 eV correspond to the lattice and defect oxygen of Bi-O and Mo-O of Bi_2_MoO_6_, respectively [46].

### 3.2. Photoelectrochemical Properties and Photocatalytic N_2_ to NH_3_

**Figure 5:**
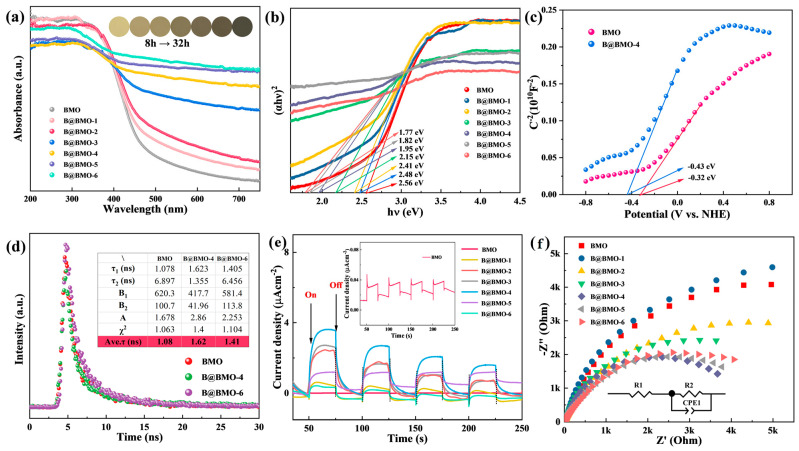
(**a**) UV-vis diffuse reflectance spectra (DRS), (**b**) band gaps of all samples, (**c**) Mott–Schottky curves of BMO and B@BMO-4, (**d**) time-resolution PL spectra, (**e**) transient photocurrent response under 300 W Xe lamp, (**f**) electrochemical impedance spectroscopy (EIS).

UV−vis DRS measurement is performed to study the light harvesting capability of the as-synthesized photocatalysts. As displayed in Figure 5a, B@BMO photocatalysts have strong visible light absorption compared with Bi_2_MoO_6_ due to the localized surface plasma resonance (LSPR) of metal Bi [47,48]. Pristine Bi_2_MoO_6_ has light absorption at 485 nm corresponding to the band gap of 2.56 eV, as shown in Figure 5b [32]. The introduction of metal Bi greatly improves the absorption efficiency for Bi_2_MoO_6_, and the colors of B@BMO photocatalysts gradually change from light yellow to dark gray (inset of Figure 5a), also suggesting the improvement in visible light absorption. The Mott–Schottky measurement is usually used to determine the conduction band minimum (CBM) position. The CBM potential of Bi_2_MoO_6_ is calculated to be −0.52 V vs. NHE which is about 0–0.2 eV more negative than the flat-band potential and that of Bi@ Bi_2_MoO_6_-4 is −0.63 V vs. NHE (Figure 5c). To reveal the charge separation efficiency, a time-resolved technique has been carried out. The electron lifetimes of the fitting results for Bi_2_MoO_6_ are 1.078 ns for *τ*_1_, 6.897 ns for *τ*_2_ and 1.08 ns for average *τ*, respectively (Figure 5d). In comparison to Bi_2_MoO_6_, B@BMO photocatalysts have a longer PL lifetime. For the B@BMO-4 composite, *τ*_1_ is 1.623 ns, *τ*_2_ is 1.355 ns and average *τ* is 1.62 ns, respectively. The long lifetime demonstrates that the Schottky junction by the introduction of metal Bi on Bi_2_MoO_6_ could effectively spur the charge separation and elongate the carrier lifetime. The measurements of photoelectrochemistry are also performed to further study the charge separation at the interface between Bi and Bi_2_MoO_6_. The transient photocurrent responses of BMO and B@BMO composites are presented in Figure 5e. The current densities of all of the samples are near zero in the dark as the reference. Once the light is turned on, a very large pulse can be observed which indicates more electrons produced. The photocurrent generated by pristine Bi_2_MoO_6_ is very low (inset of Figure 5e), while the B@BMO composites exhibit enhanced photocurrent density. Also, B@BMO-4 has the highest photocurrent among the B@BMO composites, which demonstrates that more photo-generated electrons are produced in the Schottky junction under irradiation, thereby promoting the efficient separation of core-shell B@BMO photocatalysts. The EIS is shown in Figure 5f by displaying it as Nyquist plots, which can obtain the resistance of the charges through the fitting. The diameter is small which indicates the resistance is small, which indicates an efficient charge transfer from the electrode to the solution due to the Schottky junction. The diameters of B@BMO composites are smaller than that of BMO. The impedance data are obtained through fitting the equivalent circuit. The *R*_1_, *R*_2_ and CPE represent the resistance of electrode, the resistance of transfer and the double-layer capacitance element, respectively. B@BMO-4 has the lowest *R*_2_ of about 45,032 Ω among all B@BMO photocatalysts. The results of the electrochemical measurements indicate that the core-shell Schottky junction not only promotes the separation of electrons on the semiconductor but also accelerates the interfacial transfer from Bi_2_MoO_6_ to metal Bi, just like as reported by previous articles [22].

**Figure 6:**
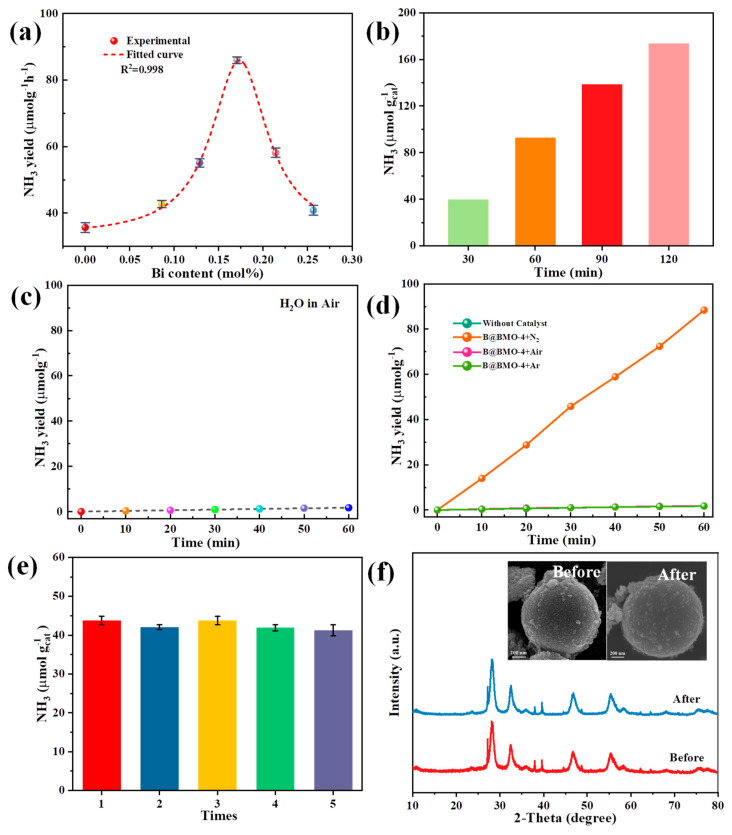
(**a**) Dependence of photocatalytic ammonia production on metallic Bi in Bi@Bi_2_MoO_6_ composites under 300 W Xe lamp irradiation, (**b**) dependence of photocatalytic ammonia production on reaction time, (**c**) blank experiment without photocatalyst, (**d**) controlled experiments for photocatalytic ammonia production over Bi@Bi_2_MoO_6_ composites under different conditions, (**e**) photostability of Bi@Bi_2_MoO_6_ photocatalysts for ammonia production and (**f**) XRD patterns and SEM images of B@BMO-4 before and after photocatalytic ammonia production.

The photoactivities of core-shell Bi@Bi_2_MoO_6_ composites for N_2_-to-ammonia conversion have been investigated using deionized water without any scavenger. The produced NH_3_ is spectrophotometrically measured based on the indophenol blue method [49]. The amount of ammonia over Bi_2_MoO_6_ and core-shell Bi@Bi_2_MoO_6_ composites with different contents of metal Bi confirmed by the ICP measurement is shown in Figure 6a. The rate of photocatalytic nitrogen to ammonia on pure Bi_2_MoO_6_ is 35.70 μmol g^−1^ h^−1^, but the generation rate of ammonia increases to 42.74 μmol g^−1^ h^−1^ when metal Bi is self-reduced onto Bi_2_MoO_6_ to Bi@ Bi_2_MoO_6_ core-shell composites (B@BMO-2) due to the increase in Bi active sites. With the increase in the Bi amount, the efficiency of the N_2_-to-ammonia conversion also increases and achieves a maximum (86.00 μmol g^−1^ h^−1^) when the content of Bi is about 0.17% (B@BMO-4 photocatalyst). While the amount of Bi increases further, the yield of the photocatalytic N_2_-to-ammonia conversion decreases. The result indicates that the activity of the photocatalytic nitrogen-to-ammonia conversion on Bi@ Bi_2_MoO_6_ core-shell composites is not a linearly dependent relationship. There is an optimal value of metal Bi. When more Bi precursors are self-reduced to metal Bi, the amount of Bi_2_MoO_6_ would decrease which results in the decrease of light absorption, thus further influencing the photocatalytic N_2_-to-ammonia conversion. However, when more Bi precursors generate a Bi_2_MoO_6_ semiconductor, the amount of Bi is very small which results in the decrease in catalytic reaction due to the lack of more Bi active sites. As a result, the activity of the photocatalytic nitrogen-to-ammonia conversion on Bi@ Bi_2_MoO_6_ core-shell composites would present a volcano-type curve. The dependence on the reaction time over the most excellent Bi@Bi_2_MoO_6_ composite (B@BMO-4) is also explored. The photocatalytic activity for ammonia conversion presents a remarkably linear enhancement (Figure 6b). Under irradiation, the B@BMO-4 photocatalyst could produce NH_3_ with a value of 173.40 μmol g^−1^ for 2 h. A comparison in NH_3_ production between Bi@ Bi_2_MoO_6_ nanospheres and some typical photocatalysts in previous reports has been summarized in Table S1. In order to confirm the source of nitrogen, the blank experiment without any photocatalyst under irradiation has also been carried out, and the result is shown in Figure 6c. It can be seen that there is a trace amount of ammonia detected as the background, which is subtracted when quantifying ammonia production. Furthermore, the other controlled experiments over Bi@Bi_2_MoO_6_ composites under different conditions are shown in Figure 6d. Under the condition without the photocatalyst, there is no ammonia detected under N_2_ and irradiation. Such a scenario is also observed under the conditions of air and Ar atmospheres. However, an obvious enhancement in the photocatalytic N_2_ reduction reaction can been seen in the Bi@Bi_2_MoO_6_ composite under the N_2_ atmosphere, which indicates that the N element is derived from incoming N_2_. To further demonstrate the stability of the photocatalyst, a longer cycle test is performed (Figure 6e). The results show that the Bi@Bi_2_MoO_6_ composite still has strong photocatalytic nitrogen fixation activity after five cycles (30 min each reaction time). In addition, the crystallinity and morphology of the photocatalyst before and after the reaction are measured (Figure 6f). The results show that the Bi@Bi_2_MoO_6_ composite has good stability, indicating that the formation of a core-shell Bi@Bi_2_MoO_6_ composite is beneficial in improving the photocatalytic performance of N_2_ to NH_3_.

### 3.3. Mechanism

**Figure 7:**
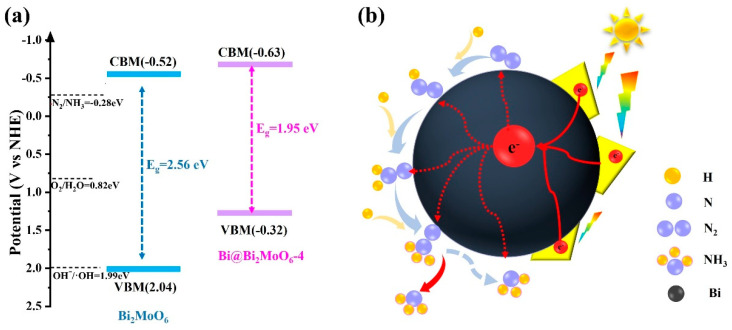
(**a**) The band structure of Bi_2_MoO_6_ and Bi@Bi_2_MoO_6_-4; (**b**) schematics of the photocatalytic N_2_-to-ammonia conversion over core-shell Bi@Bi_2_MoO_6_ photocatalysts.

The absorption wavelength of Bi_2_MoO_6_ is approximately 480 nm and that of Bi@ Bi_2_MoO_6_-4 is about 635 nm. In addition, the optical band gaps are calculated, and the Mott–Schottky measurements of Bi_2_MoO_6_ and Bi@ Bi_2_MoO_6_-4 have been performed. From the results, it can be found that the band gap energy of Bi_2_MoO_6_ is calculated from the absorption spectrum to be 2.56 eV, which is consistent with previous reports [42]. The band gap value of Bi@ Bi_2_MoO_6_-4 is about 1.95 eV, which is narrower than that of Bi_2_MoO_6_ owing to the localized surface plasmon resonance of metal Bi. The valence band maximum (VBM) potential of Bi_2_MoO_6_ is about 2.04 V vs. NHE using the equation, VBM = CBM − *E*_g_. Similarly, the VBM potential of Bi@ Bi_2_MoO_6_-4 is about 1.32 V vs. NHE. According to the results of UV-vis DRS and Mott–Schottky measurements, the schematic diagrams of the energy band arrangement of Bi_2_MoO_6_ and Bi@ Bi_2_MoO_6_-4 have been shown in Figure 7a.

Currently, there are two different associative N_2_ hydrogenation pathways, including the distal pathway and alternating pathway which are usually considered [50,51]. Some theoretical investigations reported that N_2_ favored adsorbing at Bi sites with unsaturated coordination through an end-on bound structure [35]. According to the above results and some investigations, a possible distal pathway mechanism of the photocatalytic N_2_-to-ammonia conversion over the core-shell Bi@Bi_2_MoO_6_ composite has been proposed (Figure 7b). In this distal pathway, N_2_ adsorbs on metallic Bi with the end-on bound structure. One of the N atoms on the surface is hydrogenated successively until NH_3_ is formed and released. On the basis of this pathway, only NH_3_ is produced. Actually, other nitrogen species have not been detected during photocatalytic N_2_ fixation over Bi@Bi_2_MoO_6_ photocatalysts, which further confirms the distal pathway.

## 4. Conclusions

In this work, a core-shell Bi@Bi_2_MoO_6_ photocatalyst through constructing Schottky junctions is explored for N_2_ reduction to ammonia. Metal Bi not only promotes the photo-generated electron and hole separation owing to the Schottky junction at the interface of Bi and Bi_2_MoO_6_ but also improves N_2_ adsorption and activation at Bi active sites through an end-on bound structure synchronously. As a result, the yield of the photocatalytic N_2_-to-ammonia conversion reaches up to 173.40 μmol g^−1^ on core-shell Bi@Bi_2_MoO_6_ photocatalysts, as much as two times of that of bare Bi_2_MoO_6_. This work provides a new design for the decarbonization of the nitrogen reduction reaction by the utilization of renewable energy sources.

## Data Availability

Data are contained within the article.

## Supplementary Materials

The following supporting information can be downloaded at: https://www.mdpi.com/article/10.3390/nano14090780/s1, Table S1. Comparison of photocatalytic N2 reduction activity on Bi-based photocatalysts. References [22,24,36,43,52,53,54,55,56,57,58,59,60,61,62,63,64,65] are cited in the Supplementary Materials.
