# Supplementary material for: Schottky Junctions with Bi@Bi2MoO6 Core-Shell Photocatalysts toward High-Efficiency Solar N2-to-Ammonnia Conversion in Aqueous Phase
Source: Nanomaterials (Basel). 2024 Apr 30;14(9):780. doi: 10.3390/nano14090780 (PMC11085196; doi:10.3390/nano14090780)
Supplement: Supplementary file 1 [file nanomaterials-14-00780-s001.zip › nanomaterials-2980233-supplementary.pdf]

Supplementary Material

Schottky junctions with Bi@Bi_2MoO_6 core-shell photocatalysts toward high-efficiency solar N_2-to-ammonnia conversion in aqueous phase

Meijiao Wang,[a] Guosong Wei,[a] Renjie Li,[a] Meng Yu,[a] Guangbo Liu,[b,*] Yanhua Peng[a,*]

[a]*College of Chemistry and Chemical Engineering, Qingdao University, Qingdao 266071, Shandong, P. R. China.*

[b]*Key Laboratory of Biofuels, Qingdao Institute of Bioenergy and Bioprocess Technology, Chinese Academy of Sciences, Qingdao 266101, Shandong, China*

*Corresponding Authors

Email: liugb@qibebt.ac.cn, yhpeng@qdu.edu.cn

Table S1. Comparison of photocatalytic N_2 reduction activity on Bi-based photocatalysts.

Catalyst	Light Source	NH_3/NH_4^+ Yield ($\mu mol \cdot g^{-1} \cdot h^{-1}$)	Ref.
$Bi@Bi_2MoO_6$	300 W Xe lamp	86.0	**This work**
Bi_2MoO_6-Br-Ov	300 W Xe lamp (λ≥420 nm)	32.0	[1]
BiOBr-Fe-OVs	300 W Xe lamp (λ > 400 nm)	46.1	[2]
In-Bi_2MoO_6	300 W Xe lamp (30.72 $mW \cdot cm^{-2}$)	53.4	[3]
g-C_3N_4/Bi_2MoO_6	500 W Xe lamp (λ > 420)	43.6	[4]
Bi/BiOBr	300 W Xe lamp	78.6	[5]
W-Bi_2MoO_6	Two 45 W white LED bulbs	2240	[6]
S-Bi_2MoO_6	300 W Xe lamp (λ≥420 nm)	122.1	[7]
Fe/$Bi_2O_{2.33}$	300 W Xe lamp	118.7	[8]
BiOBr/$Bi_4O_5Br_2$	300 W Xe lamp (λ≥420 nm)	66.9	[9]
In_2O_3/Bi_2MoO_6	300 W Xe lamp (30.72 $mW \cdot cm^{-2}$)	150.9	[10]
Bi_2MoO_6/Bi-MOF	300 W Xe lamp (λ≥420 nm)	125.8	[11]
$BiVO_4$/ Sv-$ZnIn_2S_4$	300 W Xe lamp (λ > 400 nm)	80.6	[12]
$Bi_{12}O_{17}Cl_2$-OVs	500 W Xe lamp	23.43	[13]

CeO_2-AD/Au	300 W Xe lamp	215.1	[14]
AgPt–TiO_2	300 W Xe lamp	38.4	[15]
Ag/AgBr-δ-Bi_2O_3	400 W Xe lamp	364.2	[16]
Au/TiO_2-Vo	300 W Xe lamp (λ≥420 nm)	78.6	[17]
Au/end-CeO_2	Semiconductor diode laser (808 nm)	114.3	[18]

Reference

[1] Wang, G.; Huo, T.; Deng, Q.; Yu, F.; Xia, Y.; Li, H.; Hou, W. Surface-layer bromine doping enhanced generation of surface oxygen vacancies in bismuth molybdate for efficient photocatalytic nitrogen fixation. *Applied Catalysis B: Environmental* **2022**, *310*, 121319, doi:10.1016/j.apcatb.2022.121319.

[2] Chen, X.; Zhang, X.; Li, Y.-H.; Qi, M.-Y.; Li, J.-Y.; Tang, Z.-R.; Zhou, Z.; Xu, Y.-J. Transition metal doping BiOBr nanosheets with oxygen vacancy and exposed {102} facets for visible light nitrogen fixation. *Applied Catalysis B: Environmental* **2021**, *281*, 119516, doi:10.1016/j.apcatb.2020.119516.

[3] Ma, T.; Yang, C.; Guo, L.; Soomro, R.A.; Wang, D.; Xu, B.; Fu, F. Refining electronic properties of Bi_2MoO_6 by In-doping for boosting overall nitrogen fixation via relay catalysis. *Applied Catalysis B: Environmental* **2023**, *330*, 122643, doi:10.1016/j.apcatb.2023.122643.

[4] Vesali-Kermani, E.; Habibi-Yangjeh, A.; Diarmand-Khalilabad, H.; Ghosh, S. Nitrogen photofixation ability of g-C_3N_4 nanosheets/Bi_2MoO_6 heterojunction photocatalyst under visible-light illumination. *Journal of Colloid and Interface Science* **2020**, *563*, 81-91, doi:10.1016/j.jcis.2019.12.057.

[5] Huang, Y.; Zhu, Y.; Chen, S.; Xie, X.; Wu, Z.; Zhang, N. Schottky Junctions with Bi Cocatalyst for Taming Aqueous Phase N_2 Reduction toward Enhanced Solar Ammonia Production. *Advanced Science* **2021**, *8*, doi:10.1002/advs.202003626.

[6] Sharma, M.; Kumar, A.; Gill, D.; Jaiswal, S.; Patra, A.; Bhattacharya, S.; Krishnan, V. Boosting Photocatalytic Nitrogen Fixation via Nanoarchitectonics Using Oxygen Vacancy Regulation in W-Doped Bi_2MoO_6 Nanosheets. *ACS Applied Materials & Interfaces* **2023**, *15*, 55765-55778, doi:10.1021/acsami.3c12563.

[7] Liu, Z.; Luo, M.; Cao, Y.; Meng, L.; Yang, Y.; Li, X. Tuning the electronic properties of Bi_2MoO_6 by S-doping to boost efficient photocatalytic nitrogen fixation reactions. *Journal of Catalysis* **2024**, *430*, doi:10.1016/j.jcat.2024.115347.

[8] Zhu, C.; Zhang, L.; Cui, L.; Zhang, Z.; Li, R.; Wang, Y.; Wang, Y.; Fan, C.; Yu, Z.; Liu, J. Fe-Bi dual sites regulation of $Bi_2O_{2.33}$ nanosheets to promote photocatalytic nitrogen fixation activity. *Journal of Colloid and Interface Science* **2024**, *661*, 46-58, doi:10.1016/j.jcis.2024.01.082.

[9] Wang, H.; Chen, Z.; Shang, Y.; Lv, C.; Zhang, X.; Li, F.; Huang, Q.; Liu, X.; Liu, W.; Zhao, L.; et al. Boosting Carrier Separation on a BiOBr/$Bi_4O_5Br_2$ Direct Z-Scheme Heterojunction for Superior Photocatalytic Nitrogen Fixation. *Acs Catalysis* **2024**, doi:10.1021/acscatal.3c06169.

[10] Huang, X.; Du, R.; Zhang, Y.; Ren, J.; Yang, Q.; Wang, K.; Ni, Y.; Yao, Y.; Ali Soomro, R.; Guo, L.; et al. Modulating charge oriented accumulation via interfacial chemical-bond on In_2O_3/Bi_2MoO_6

heterostructures for photocatalytic nitrogen fixation. *Journal of colloid and interface science* **2024**, *664*, 33-44, doi:10.1016/j.jcis.2024.03.018.
[11] Dong, Q.; Li, X.; Sun, J.; Zhu, Y.; Liang, X.; Ren, H.; Labidi, A.; Wang, D.; Li, F.; Wang, C. Regulating concentration of surface oxygen vacancies in Bi_2MoO_6/Bi-MOF for boosting photocatalytic ammonia synthesis. *Journal of Catalysis* **2024**, *433*, doi:10.1016/j.jcat.2024.115489.
[12] Zhang, G.; Yuan, X.; Xie, B.; Meng, Y.; Ni, Z.; Xia, S. S vacancies act as a bridge to promote electron injection from Z-scheme heterojunction to nitrogen molecule for photocatalytic ammonia synthesis. *Chemical Engineering Journal* **2022**, *433*, doi:10.1016/j.cej.2021.133670.
[13] Kok, S.H.W.; Lee, J.; Chong, W.K.; Ng, B.-J.; Kong, X.Y.; Ong, W.J.; Chai, S.-P.; Tan, L.-L. Bismuth-rich $Bi_{12}O_{17}Cl_2$ nanorods engineered with oxygen vacancy defects for enhanced photocatalytic nitrogen fixation. *Journal of Alloys and Compounds* **2023**, *952*, doi:10.1016/j.jallcom.2023.170015.
[14] Li, H.; Zhang, J.; Deng, X.; Wang, Y.; Meng, G.; Liu, R.; Huang, J.; Tu, M.; Xu, C.; Peng, Y.; et al. Structure and Defect Engineering Synergistically Boost High Solar-to-Chemical Conversion Efficiency of Cerium oxide/Au Hollow Nanomushrooms for Nitrogen Photofixation. *Angewandte Chemie-International Edition* **2024**, *63*, doi:10.1002/anie.202316384.
[15] Bian, X.; Zhao, Y.; Zhang, S.; Li, D.; Shi, R.; Zhou, C.; Wu, L.-Z.; Zhang, T. Enhancing the Supply of Activated Hydrogen to Promote Photocatalytic Nitrogen Fixation. *Acs Materials Letters* **2021**, *3*, 1521-1527, doi:10.1021/acsmaterialslett.1c00504.
[16] Gao, X.; Shang, Y.; Liu, L.; Gao, K. Ag plasmon resonance promoted 2D AgBr-δ-Bi_2O_3 nanosheets with enhanced photocatalytic ability. *Journal of Alloys and Compounds* **2019**, *803*, 565-575, doi:10.1016/j.jallcom.2019.06.311.
[17] Yang, J.; Guo, Y.; Jiang, R.; Qin, F.; Zhang, H.; Lu, W.; Wang, J.; Yu, J.C. High-Efficiency "Working-in-Tandem" Nitrogen Photofixation Achieved by Assembling Plasmonic Gold Nanocrystals on Ultrathin Titania Nanosheets. *Journal of the American Chemical Society* **2018**, *140*, 8497-8508, doi:10.1021/jacs.8b03537.
[18] Jia, H.; Du, A.; Zhang, H.; Yang, J.; Jiang, R.; Wang, J.; Zhang, C.-y. Site-Selective Growth of Crystalline Ceria with Oxygen Vacancies on Gold Nanocrystals for Near-Infrared Nitrogen Photofixation. *Journal of the American Chemical Society* **2019**, *141*, 5083-5086, doi:10.1021/jacs.8b13062.
